# Loudspeaker cabinet design by topology optimization

**DOI:** 10.1038/s41598-023-46170-4

**Published:** 2023-12-01

**Authors:** Ahmad H. Bokhari, Martin Berggren, Daniel Noreland, Eddie Wadbro

**Affiliations:** 1 Department of Computing Science, Umeå University, 901 87 Umeå, Sweden; 2 The Forestry Research Institute of Sweden (Skogforsk), Uppsala Science Park, 75183 Uppsala, Sweden; 3 Department of Mathematics and Computer Science, Karlstad University, 651 88 Karlstad, Sweden

**Keywords:** Applied mathematics, Acoustics, Computational science

## Abstract

Using material distribution-based topology optimization, we optimize the bandpass design of a loudspeaker cabinet targeting low frequencies. The objective is to maximize the loudspeaker’s output power for a single frequency as well as a range of frequencies. To model the loudspeaker’s performance, we combine a linear electromechanical transducer model with a computationally efficient hybrid 2D–3D model for sound propagation. The adjoint variable approach computes the gradients of the objective function with respect to the design variables, and the Method of Moving Asymptotes (MMA) solves the topology optimization problem. To manage intermediate values of the material indicator function, a quadratic penalty is added to the objective function, and a non-linear filter is used to obtain a mesh independent design. By carefully selecting the target frequency range, we can guide the optimization algorithm to successfully generate a loudspeaker design with the required bandpass character. To the best of our knowledge, this study constitutes the first successful attempt to design the interior structure of a loudspeaker cabinet using topology optimization.

## Introduction

Loudspeaker systems^1,2^ are designed to reproduce sound within the range of human hearing. However, it is difficult to design a single loudspeaker that can efficiently reproduce the whole frequency range. Therefore, separate loudspeakers are often used to cover different parts of the sound spectrum. The loudspeaker aimed at the lowest frequencies is referred to as a subwoofer. Public address systems, movie theaters, home theaters, and car audio systems all use subwoofers. Here, we optimize a so-called bandpass^3,4^ design of a subwoofer loudspeaker, in which a transducer is mounted in a sealed back chamber and radiates into a ported front chamber. The back chamber serves as a high pass filter, while the front chamber and port serve as a low pass filter, jointly forming an acoustic bandpass filter. The high pass filter restricts the movement of the transducer’s membrane and protects it from exceeding excursion limits. The low pass filter hampers the transmission of high frequencies, including spurious ones caused by distortion in the transducer, an effect not possible to achieve with mere signal processing at the source. This type of loudspeaker can be designed for a narrow frequency band with high efficiency or a wider frequency band that also includes the low frequencies at the expense of efficiency.

In this study, we use a material distribution based topology optimization method to design the loudspeaker cabinet. This method seeks to the optimal placement of material inside a design domain. This domain is divided into pixels (or voxels in 3D), and an optimization algorithm finds for each pixel whether or not it should be occupied by material to extremize an objective function. Initial work by Bendsøe and Kikuchi^5^ laid the foundation for material distribution based topology optimization. Bendsøe and Sigmund^6^ comprehensively summarize early research on topology optimization techniques and their applications. This concept has been successfully employed for optimizing modern automotive^7^ and aircraft structures^8,9^. Moreover, the method has also proven successful for other applications, such as fluid flow^10^, heat transfer^11^, optics^12^, electromagnetics^13,14^, and acoustics^15–17^; however, techniques for design optimization are still maturing in these fields. The methods in acoustics focus on optimizing individual components, such as loudspeaker horns^15^ and sound mufflers^18,19^, under idealized conditions. However, real-life acoustic systems consist of many components that interact and affect each other’s performance. Separate optimization of each system component under idealized conditions will most likely yield a sub-optimal design. In recent studies, topology optimization has been used to optimize phase plugs^20^, waveguides^21^, as well as material properties of a transducer^22^. In this study, we do not aim to optimize a transducer or elements of loudspeaker. Instead, we employ topology optimization to optimize the loudspeaker cabinet by using a model that takes into account the transducer’s effects within the linear regime.

## Acoustic modeling

**Figure 1:**
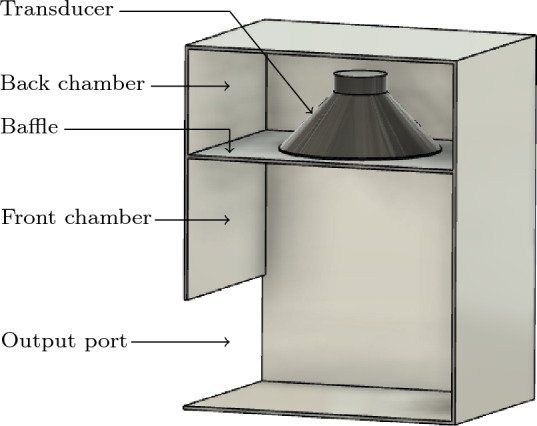
Cutaway drawing of the loudspeaker with an empty front chamber.

Consider the loudspeaker setup illustrated in Fig. 1. A transducer is mounted in a sealed back chamber at the center of a baffle and an output port is located in the front chamber. We will see that this base design indeed possesses a bandpass quality, albeit quite poorly. To improve the performance characteristics, a material distribution based topology optimization algorithm will place additional solid material within the front chamber. To model the sound propagation from the applied voltage on the voice coil of the transducer, through the loudspeaker’s interior, and out to the exterior, we developed a full 3D as well as a computationally efficient hybrid model^23^ that uses a modular approach where the properties of interacting modules can be precomputed. The 3D model is not computationally feasible to use in an optimization loop. Hence, we employ the computationally more efficient hybrid method in the optimization loop, and validate the final result using the 3D model.

In the 3D as well as hybrid method, we employ lumped-element models of the electrical as well as mechanical properties of the moving coil transducer. Furthermore, we use a stiff approximation of the transducer’s diaphragm. Thus, the models are restricted to the small-signal regime. The lumped equations are coupled to the finite element model, which solves for the acoustic pressure inside the loudspeaker. In addition, we employ the boundary element method to model the interaction of the loudspeaker’s port with the exterior.

To generate sound at low frequencies, the transducer needs to move large air volumes, which can cause over excursion, moving the coil to a region of non-homogeneous flux density, resulting in a non-linear behavior. We do not aim to model this or other non-linear effects that can generate distortion in the form of high harmonics. However, we note that in the bandpass design, the front chamber dampens high harmonic distortion induced by the transducer, while the back chamber limits the excursion by providing additional stiffness to the transducer. Moreover, the improved performance also reduces the required excursion for a given sound pressure and consequently distortion. Hence, the bandpass design of loudspeaker counteracts non-linearities in the transducer. Other complex behaviors, such as modal break up of the diaphragm movement, is of little concern here, because the diaphragm essentially acts as a piston at low frequencies; the first modal break up occurs at frequencies above $700\,\textrm{Hz}$ for conventional diaphragms^2^, p. 15. Another important aspect in designing a loudspeaker aimed at low frequencies is to select cabinet material that is acoustically rigid. High sound pressures can cause resonances in the walls if they are not thick enough, causing coloring and distortion of sound. The most common materials for building loudspeaker enclosures are plywood and MDF (medium-density fiberboard), which have good damping properties. The wall thickness varies depending on the cabinet volume and the size of the transducer. We assume that the walls of the loudspeaker enclosure are thick enough to be acoustically rigid. In the following two sections, we present a summary of the 3D and the hybrid model, respectively. For details, we refer to the full account^23^.

## The 3D model

**Figure 2:**
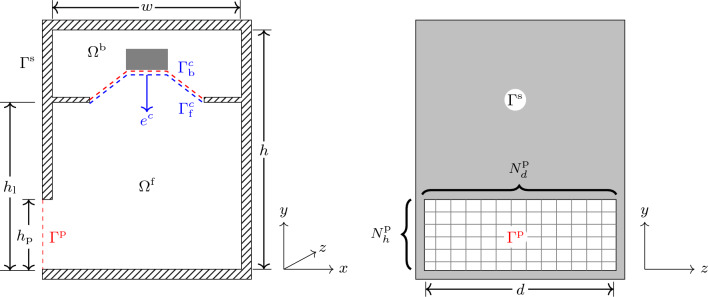
The 3D model. Left: Cross-section of the loudspeaker with an empty front chamber. Right: Front view of the loudspeaker where $\Gamma ^{\text {p}}$ is divided into $N^{\text {p}}=N^{\text {p}}_h\times N^{\text {p}}_d$ panels.

Consider the cross-section of the loudspeaker setup illustrated in Fig. 2 (left), with dimensions $w\times h\times d$, where *d* is in a direction perpendicular to the plane. The dimensions of the baffle and the output port are $w \times d$ and $h_{\text {p}} \times d$, respectively. Let $\Gamma ^c_\text {b}$ and $\Gamma ^c_{\text {f}}$ denote the back and the front of the transducer’s diaphragm, respectively. Similarly, let $\Omega ^\text {b}$ and $\Omega ^{\text {f}}$ denote the back and the front chamber, respectively. Let $e^c$ be the unit vector in the direction of motion of diaphragm, the negative *y*-direction in Fig. 2 (left). Moreover, let $\Gamma ^{\text {p}}$ denote the output port that separates the loudspeaker’s interior from its exterior, and $\Gamma ^\text {s}$ all sound-hard walls.

### The loudspeaker’s interior

We consider time-harmonic linear wave propagation inside the loudspeaker’s interior and assume that the acoustic pressure satisfies $P(\varvec{x},t) = \Re \{p(\varvec{x}) e^{{\textrm{i}}\omega t}\}$, where $\textrm{i}$ is the imaginary unit, *p* the complex pressure amplitude, $\omega$ the angular frequency, and *t* the time. This assumption gives us the Helmholtz equation for *p*. That is,

1 $$\begin{aligned} \omega ^2 p + c^2 \Delta p = 0, \quad \text {in } \Omega ^{\text {f}}\cup \Omega ^\text {b}, \end{aligned}$$

where *c* is the speed of sound and $\Delta = \nabla \cdot \nabla$ the Laplace operator.

As stated earlier, we assume all the walls to be rigid (sound-hard), which implies the boundary condition

2 $$\begin{aligned} \frac{\partial p}{\partial n} = 0, \quad \text {on } \Gamma ^\text {s}. \end{aligned}$$

The linearized Euler equation gives a relation between the diaphragm velocity $u^{\text {c}}$ and the pressure $p^\text {b}$ inside $\Omega ^\text {b}$,

3a $$\begin{aligned} \frac{\partial p^{\text{b}}}{\partial n^{\text{b}}} + {\textrm{i}}k\rho c u^{\text{c}} e^{\text {c}} \cdot n^{\text{b}} = 0 \quad {\text{on }} \Gamma ^{\text {c}}_{\text {b}}, \end{aligned}$$

and the pressure $p^{\text {f}}$ inside $\Omega ^{\text {f}}$,

3b $$\begin{aligned} \frac{\partial p^{\text {f}}}{\partial n^{\text {f}}} + {\textrm{i}}k\rho c u^{\text {c}} e^{\text {c}} \cdot n^{\text {f}} =0 \quad {\text {on }} \Gamma ^{\text {c}}_{\text {f}}, \end{aligned}$$

where $k=\omega /c$ is the wave number, $\rho$ is the air density, and $n^\text {b}$ and $n^{\text {f}}$ are outward directed normals with respect to $\Omega ^\text {b}$ and $\Omega ^{\text {f}}$, respectively.

Furthermore, we use mechanical and electric circuit equations to model the electromechanical properties of the transducer. The mechanical model expresses the balance of forces on the speaker diaphragm,

4 $$\begin{aligned} \left( -\omega ^2 M_{\text{md}} + {\textrm{i}} \omega R_{\text {ms}} + \frac{1}{C_{\text {ms}}}\right) u^{\text {c}} = {\textrm{i}} \omega \left[ BlI + \int _{\Gamma ^{\text {c}}} e^{\text {c}}\cdot (n^{\text {b}} p^{\text {b}} + n^{\text {f}} p^{\text {f}})\right] , \end{aligned}$$

where $M_\text {md}$ is the moving mass of the diaphragm, $R_{\text {ms}}$ the mechanical resistance, $C_{\text {ms}}$ the mechanical compliance, *Bl* the force factor, and *I* the electric current amplitude in the voice coil.

#### Remark 1

Throughout this article, we omit symbols of measure, such as $d\Gamma$ or $d\Omega$, in integral expressions, since the type of measure will be clear from the domain of integration.

**Figure 3:**
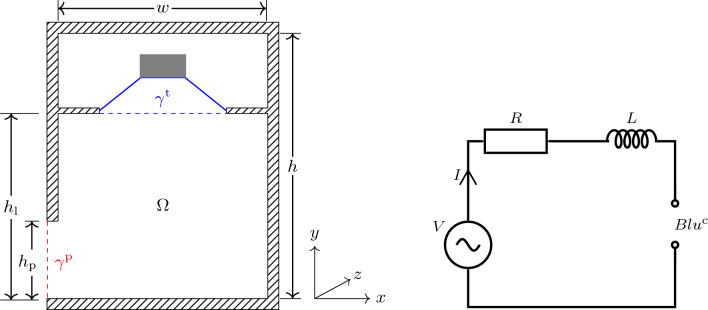
Left: The hybrid model, cross-section of loudspeaker with an empty front chamber. Right: The electric circuit model of the transducer.

To complete the transducer model, we use the simple electric circuit illustrated in Fig. 3 (right) given by

5 $$\begin{aligned} (R+{\textrm{i}}\omega L)I + Bl u^{\text {c}} = V, \end{aligned}$$

where the amplifier voltage *V* is the input to the system, *R* is the electric resistance, and *L* is the inductance.

### The loudspeaker’s exterior

To model the interaction of the output port $\Gamma ^{\text {p}}$ with the loudspeaker’s exterior, we divide $\Gamma ^{\text {p}}$ into $N^{\text {p}}=N^{{\text {p}}}_h \times N^{{\text {p}}}_d$ square panels, $\Gamma ^{\text {p}}_j$, where $j = 1,2,\ldots ,N^{\text {p}}$, as illustrated in Fig. 2 (right). For each panel, we let $u^{\text {p}}_j$ denote the complex normal velocity on $\Gamma ^{\text {p}}_j$ and $\langle p\rangle _{\Gamma ^{\text {p}}_j}$ the average pressure on $\Gamma ^{\text {p}}_j$. We assemble an $N^{\text {p}}\times N^{\text {p}}$ matrix $\varvec{Z}^{\text {p}}_\text {3D}$ that relates the normal velocities $\varvec{u}^{\text {p}} = \big [u^{\text {p}}_1, u^{\text {p}}_2,\ldots , u^{\text {p}}_{N^{\text {p}}}\big ]^T$ to the pressures $\varvec{p}^{\text {p}} = \big [\langle p\rangle _{\Gamma ^{\text {p}}_1},\langle p\rangle _{\Gamma ^{\text {p}}_2}, \ldots , \langle p\rangle _{\Gamma ^{\text {p}}_{N^{\text {p}}}} \big ]^T$ through the impedance relation

6 $$\begin{aligned} \varvec{Z}^{\text {p}}_\text {3D} \varvec{u}^{\text {p}} = \varvec{p}^{\text {p}}. \end{aligned}$$

For each frequency under consideration, we compute the impedance matrix $\varvec{Z}^{\text {p}}_\text {3D}$ column by column by solving $N^{\text {p}}$ exterior Helmholtz problems with successive unit excitations on each of the panels. The boundary-element solver in the commercial Pafec VibroAcoustics software is used for this calculation.

## The hybrid model

Consider the cross-section of the loudspeaker illustrated in Fig. 3 (left). For computational purposes, we split the interior into two domains at the baffle, an upper box formed by the domain above the baffle, and a lower box, with dimensions $w\times h_\text {l}\times d$, formed by the domain below the baffle. The domain $\Omega$ is the full lower box. We assume that all the walls and internal solid structures inside $\Omega$ are extruded in the *z*-direction. Let $\gamma ^{\text {t}}$ denote the boundary that separates the two domains, and let $\gamma ^{\text {p}}$ denote the output port.

We assume planar symmetry in the acoustic pressure along the *z*-axis in the air region of $\Omega$. The variations in the direction of the *z*-axis will be negligible due to the long wavelengths. This assumption allows the use of 2D wave propagation (in the *xy*-plane), which provides two advantages. First, it is computationally advantageous for use in an optimization loop compared to a full 3D model. Second, the planar symmetry provides construction advantages, as the interior can then be built by placing, say, wooden slabs aligned with the *z*-axis in the lower box. However, we employ a 3D model for the upper box and the exterior because 3D effects cannot be avoided there.

### The loudspeaker’s exterior

We assume planar symmetry in the lower box for the acoustic pressure, but we cannot use the same assumption to model the interaction with the exterior. Similarly, as for the 3D model, we pre-compute the acoustic properties by assembling an impedance matrix. We assume the acoustic velocity to be constant on each boundary segment $\gamma ^p_j$, $j=1, \dots , N_h^p$ and extend each of these into depth-running strips, $\gamma ^{\text {p}}_j\times (0,d)$ on which the acoustic velocity still is assumed to be constant. Now the exterior response of a unit velocity on each strip can be computed using the full 3D boundary-element method. In fact, the response is already available from the matrix $\varvec{Z}^{\text {p}}_\text {3D}$ by adding all columns corresponding to a particular strip. For each strip on the port, averaging corresponding rows of $\varvec{Z}^{\text {p}}_\text {3D}$, we obtain the average pressure response to the applied unit strip velocity. In this way, we obtain an $N^{\text {p}}_h\times N^{\text {p}}_h$ matrix $\varvec{Z}^{\text {p}}_\text {2D}$ that relates the normal velocities $\varvec{u}^{\text {p}}$ on each strip $\gamma ^{\text {p}}_j\times (0,d)$ to the average pressures $\varvec{p}^{\text {p}}$ on all strips through the impedance relation

7 $$\begin{aligned} \varvec{Z}^{\text {p}}_\text {2D} \varvec{u}^{\text {p}} = \varvec{p}^{\text {p}}, \end{aligned}$$

where $\varvec{u}^{\text {p}}= \big [u^{\text {p}}_1, u^{\text {p}}_2,\ldots , u^{\text {p}}_{N^{\text {p}}_h}\big ]^T$ and $\varvec{p}^{\text {p}} = \big [\langle p\rangle _{\gamma ^{\text {p}}_1},\langle p\rangle _{\gamma ^{\text {p}}_2},$ $\ldots , \langle p\rangle _{\gamma ^{\text {p}}_{N^{\text {p}}_h}} \big ]^T$, in which $\langle p\rangle _{\gamma ^{\text {p}}_j}$ and $u^{\text {p}}_j$ holds the average pressure and the normal velocity on $\gamma ^{\text {p}}_j$, respectively.

### The loudpeaker’s interior

#### The upper box

We seek to represent the interaction at $\gamma ^{\text {t}}$ with the upper box in terms of the acoustic response of the back chamber as well as the coupling to the electromechanical model of the transducer. Hence, in addition to pressures and velocities, also the diaphragm velocity $u^{\text {c}}$ (in the negative *y*-direction) and the voice coil current *I* are taken into account while computing the response of the upper box. Although it is reasonable to assume planar symmetry and thus carry out 2D calculations in the lower box, we cannot make this assumption above $\gamma ^{{\text {t}}}$ due to the presence of the cylindrically-shaped transducer that creates a local sound field extending throughout the back chamber. Therefore we make a “hybrid” ansatz and compute in full 3D in the upper box, but average the pressure response in the depth direction as it concerns the effects on the lower box.

More precisely, we divide the boundary $\gamma ^{\text {t}}$ into line segments $\gamma ^{\text {t}}_j$, where $j=1,2,\ldots ,N^{\text {t}}$, extend these into the depth region to obtain strips $\gamma ^{{\text {t}}}\times (0,d)$. Each such strip, in succession, is given a unit velocity amplitude, while the other strips, as well as the diaphragm velocity, are held at zero velocity. In addition, a unit diaphragm velocity is imposed, while the strips are held at a zero velocity. In each of these cases, the acoustic pressure is then computed in the full 3D back chamber. Finally, for each of these excitations, we compute the voice coil current *I* as well as the pressure response averaged over each of the $N^{{\text {t}}}$ strips. This procedure enables us to obtain an impedance relation of the form

8 $$\begin{aligned} \underbrace{ \begin{bmatrix} \varvec{Z}^{\text {tt}} \varvec{Z}^\text {tc} \\ \varvec{Z}^{\text {ct}} {Z}^{\text {cc}} \end{bmatrix}}_{\varvec{Z}^{\text {t}}} \begin{bmatrix} \varvec{u}^{\text {t}}\\ u^{\text {c}} \end{bmatrix} = \begin{bmatrix} \varvec{p}^{\text {t}} \\ I \end{bmatrix}, \end{aligned}$$

where the $N^{\text {t}}\times N^{\text {t}}$ block $\varvec{Z}^{\text {tt}}$ represents the acoustic response of the back chamber, the $N^{\text {t}}\times 1$ block $\varvec{Z}^\text {tc}$, the $1\times N^{\text {t}}$ block $\varvec{Z}^{\text {ct}}$, the $1\times 1$ block $Z^{\text {cc}}$ represent the interaction with the speaker diaphragm; $\varvec{u}^{\text {t}}= \big [u^{\text {t}}_1, u^{\text {t}}_2,\ldots , u^{\text {t}}_{N^{\text {t}}}\big ]^T$ and $\varvec{p}^{\text {t}} = \big [\langle p\rangle _{\gamma ^{\text {t}}_1},\langle p\rangle _{\gamma ^{\text {t}}_2}, \ldots , \langle p\rangle _{\gamma ^{\text {p}}_{N^{\text {t}}}} \big ]^T$, in which $u^{\text {t}}_j$ is the normal velocity on $\gamma ^{\text {t}}_j$, and $\langle p\rangle _{\gamma ^{\text {t}}_j}$ denotes the average pressure on $\gamma ^{\text {t}}_j$. To compute this matrix, we set up a full 3D finite element model of the upper box, including a linear electromechanical model of the transducer, in the commercial software Comsol Multiphysics. Matrix $\varvec{Z}^{\text {t}}$ is then computed, for each frequency, column by column by exciting the velocity of each strip in succession as well as an excitation of the diaphragm and computing corresponding voice coil current and averaging the pressure response over each strip.

#### The lower box

**Figure 4:**
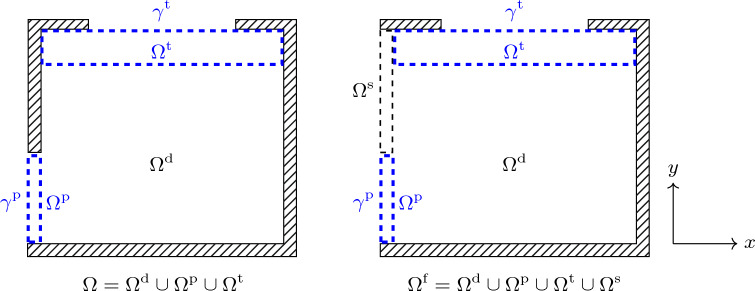
Left: Computational domain $\Omega$. Right: Filtering domain $\Omega ^{\text {f}}$.

Physically, the panels $\gamma ^{\text {p}}_j$ and $\gamma ^{\text {t}}_j$ correspond to massless and stiff pistons. The acoustic impedance relation is valid provided that the boundaries $\gamma ^{\text {p}}$ and $\gamma ^{\text {t}}$ have air on both sides. To ensure this property, we split $\Omega$ into three non-intersecting parts, illustrated in Fig. 4, denoted $\Omega ^{\text {t}}$, $\Omega ^{\text {d}}$, and $\Omega ^{\text {p}}$ and do not allow material to be placed in $\Omega ^{\text {t}}$ and $\Omega ^{\text {p}}$.

Further, we define material indicator function $\alpha$ such that $\alpha =1$ in the air region and $\alpha =0$ in the solid region. (In practice, we use $\alpha =1$ in the air region and $\alpha =\varepsilon >0$ in the solid, where $\varepsilon$ is a small positive number.) As mentioned earlier, we do not allow material to be placed in $\Omega ^{\text {t}}$ and $\Omega ^{\text {p}}$. That is, we require $\alpha \equiv 1$ in both $\Omega ^{\text {p}}$ and $\Omega ^{\text {t}}$. Wave propagation in the lower box is governed by the following Helmholtz equation for the acoustic pressure,

9 $$\begin{aligned} \omega ^2 \alpha p + c^2\nabla \cdot (\alpha \nabla p) = 0 \hspace{5.0pt}\text {in } \Omega . \end{aligned}$$

##### Remark 2

The model in Eq. (9), where the material indicator function $\alpha$ controls the presence and absence of material inside $\Omega$, was introduced by Wadbro and Berggren^15^ for acoustic horn optimization and has been used in many contributions since then. In material distribution topology optimization, redefining the material indicator function by replacing $\alpha = 0$ with $\alpha = \varepsilon >0$ is a standard strategy^6^ to obtain a unique solution to the governing equation. A relevant question is how much this approximation will affect the solution. Using this approximation in the Helmholtz type Eq. (9), Kasolis et al.^24^ performed an analysis which revealed that the error is linear in $\varepsilon$ to the solution of an exactly modeled scatterer with Neumann conditions on the sound-hard walls. In the computations reported below, we set $\varepsilon =10^{-3}$.

### Finite element discretization

The domain $\Omega$ is discretized using a uniform mesh of square elements. We use standard continuous, piecewise bi-quadratic basis functions for the complex pressure, denoted by $\varphi _1,\,\varphi _2, \ldots ,\,\varphi _{N^{\Omega }}$, where $N^\Omega$ is the number of degrees of freedom in the finite element approximation. In addition, we approximate $\alpha$ by an element-wise constant material indicator function $\alpha _h$.

By putting together a finite element discretized version of the governing Eq. (9), impedance relations (8) and (7), and circuit Eq. (5), we arrive at the following equation system

10 $$\begin{aligned} \underbrace{\begin{bmatrix} {\varvec{A}}^{\text {ll}} &{} {\varvec{A}}^{\text {lp}} &{} {\varvec{A}}^{\text {lt}} &{} \varvec{0} &{} \varvec{0} \\ {\varvec{A}}^{\text {pl}} &{} \varvec{Z}^{\text {p}} &{} \varvec{0} &{} \varvec{0} &{} \varvec{0} \\ {\varvec{A}}^{\text {tl}} &{} \varvec{0} &{} \varvec{Z}^{\text {tt}} &{} \varvec{Z}^\text {tc} &{} \varvec{0} \\ \varvec{0} &{} \varvec{0} &{} \varvec{Z}^{\text {ct}} &{} Z^{\text {cc}} &{} -1 \\ \varvec{0} &{} \varvec{0} &{} \varvec{0} &{} a^\text {Ic} &{} a^\text {II} \end{bmatrix}}_{{\varvec{A}}} \underbrace{\begin{bmatrix} \varvec{p}\\ \varvec{u}^{\text {p}}\\ \varvec{u}^{\text {t}}\\ u^{\text {c}}\\ I \end{bmatrix}}_{\varvec{x}} = \underbrace{\begin{bmatrix} 0\\ 0\\ 0\\ 0\\ V \end{bmatrix}}_{\varvec{b}}, \end{aligned}$$

where the entry $p_j$ in vector $\varvec{p}$ represents the complex pressure amplitude on the *j*th degree of freedom, and the $N^\Omega \times N^{\Omega }$ matrix ${\varvec{A}}^{\text {ll}}$, the $N^\Omega \times N^{\text {p}}$ matrix ${\varvec{A}}^{\text {lp}}$, the $N^\Omega \times N^{\text {t}}$ matrix ${\varvec{A}}^{\text {lt}}$, the $N^{\text {p}}\times N^\Omega$ matrix ${\varvec{A}}^{\text {pl}}$, and the $N^{\text {t}}\times N^\Omega$ matrix ${\varvec{A}}^{\text {tl}}$ have entries

11a $$\begin{aligned}&a^{\text {ll}}_{ij} =c^2 \int _{\Omega } \alpha _h \nabla \varphi _i \cdot \nabla \varphi _j -\omega ^2\int _{\Omega } \alpha _h \varphi _i \varphi _j, \end{aligned}$$

11b $$\begin{aligned}&a^{\text {lp}}_{ij} = -\textrm{i} \omega c \int _{\gamma ^{\text {p}}_{j}} \varphi _i,\quad a^{\text {lt}}_{ij} =-\textrm{i}\omega c \int _{\gamma ^{\text {t}}_{j}} \varphi _i. \end{aligned}$$

11c $$\begin{aligned}&a^{\text {pl}}_{ij} = \int _{\gamma ^{\text {p}}_{j}}\varphi _i. \end{aligned}$$

11d $$\begin{aligned}&a^{\text {tl}}_{ij} = \frac{d}{2}\int _{\gamma ^{\text {t}}_{j}} \varphi _i, \end{aligned}$$

respectively, and finally $a^\text {Ic}=Bl/(\rho c)$ and $a^{\text{II}} = R+{\textrm{i}}\omega L$.

## Design definition by filtering

To enable gradient-based optimization, we relax $\alpha _h$ to allow intermediate values that neither represent solid material nor air. We employ a combination of a penalization and an appropriate filtering approach to enforce extreme values of $\alpha _h$ and ensure size control. In this section, we detail our definition and handling of the design variables using a non-linear filtering method. The morphological dilate and erode operators are approximated by these non-linear filters. More precisely, to use gradient-based optimization, we approximate the morphological operators with the harmonic-mean based filters suggested by Svanberg and Svärd^25^, and we use the *fW*-mean based filtering framework of Wadbro and Hägg^26^.

For the optimization, $\Omega ^{\text {d}}$ is our design domain. We define the set of admissible design variables as

12 $$\begin{aligned} \mathscr {A} = \left\{ {\varvec{d}}\in \mathbb {R}^{N^{\text {d}}} \mid 0\le d_j \le 1 \right\} , \end{aligned}$$

where ${\varvec{d}}$ is a $N^{\text {d}}\times 1$ vector that defines the material distribution inside $\Omega ^{\text {d}}$ before filtering.

For the filtering, we use the extended domain $\Omega ^{\text {f}}= \Omega ^{\text {d}} \cup \Omega ^\text {s}\cup \Omega ^{\text {a}}$ illustrated in Fig. 4 (right). Here, $\Omega ^\text {s}$ is a domain occupied by solid, and $\Omega ^{\text {a}}=\Omega ^{\text {t}}\cup \Omega ^{\text {p}}$ is a domain occupied by air. The uniform mesh of square elements is extended to $\Omega ^{\text {f}}$ using elements of the same size as those in $\Omega$. The *N* elements of $\Omega ^{\text {f}}$ are sorted so that those in $\Omega ^{\text {d}}$ come first, $\Omega ^\text {s}$ second, and $\Omega ^{\text {a}}$ last.

We assemble a weight matrix $\varvec{W}_r = \varvec{D}_r^{-1}\varvec{G}_r$, where $\varvec{G}_r$ is a neighborhood indicator matrix with entries

13 $$\begin{aligned} g_{ij}= {\left\{ \begin{array}{ll} 1&{} \quad \text {if} \; \left\| x_i - x_j \right\| \le r, \\ 0&{} \quad \text {else}, \end{array}\right. } \end{aligned}$$

and $\varvec{D}_r = \text {diag}(\varvec{G}_r \varvec{1}_N )$, where $\varvec{1}_N=\left( 1,1,\ldots ,1\right) ^T\in \mathbb {R}^{\text {N}}$. In expression (13), $x_i$ and $x_j$ are the centroids of elements *i* and *j* in $\Omega ^{\text {f}}$, respectively, and $\left\| x_i - x_j \right\|$ is the distance between $x_i$ and $x_j$. In addition, we define the functions

14 $$\begin{aligned} f_{\mathscr {E}_{\beta }}(x) = (x+\beta )^{-1} \quad \text {and} \quad f_{\mathscr {D}_{\beta }}(x) = f_{\mathscr {E}_{\beta }}(1-x), \end{aligned}$$

where $\beta > 0$ is a parameter. We denote the inverse functions of $f_{\mathscr {E}_{\beta }}$ and $f_{\mathscr {D}_{\beta }}$ by $f^{-1}_{\mathscr {E}_{\beta }}$ and $f^{-1}_{\mathscr {D}_{\beta }}$, respectively.

Now, we define the discrete harmonic erode and dilate operators that act on an $N\times 1$ vector $\varvec{\eta }$ with entries $0\le \eta _N \le 1$ as

15 $$\begin{aligned} \mathscr {E}_{r,\beta }(\varvec{\eta }) = \varvec{f}^{-1}_{\mathscr {E}_{\beta }} \big (\varvec{W}_r \varvec{f}_{\mathscr {E}_{\beta }} (\varvec{\eta }) \big ) \quad \text {and} \quad \mathscr {D}_{r,\beta }(\varvec{\eta }) = \varvec{f}^{-1}_{\mathscr {D}_{\beta }} \big (\varvec{W}_r \varvec{f}_{\mathscr {D}_{\beta }}(\varvec{\eta }) \big ), \end{aligned}$$

respectively. Here, $\varvec{f}_{\mathscr {E}_{\beta }} = \big [f_{\mathscr {E}_{\beta }}(\eta _1),\,f_{\mathscr {E}_{\beta }}(\eta _2),\ldots ,f_{\mathscr {E}_{\beta }}(\eta _N) \big ]^T$ and $\varvec{f}_{\mathscr {D}_{\beta }}=\big [f_{\mathscr {D}_{\beta }}(\eta _1),$ $\,f_{\mathscr {D}_{\beta }}(\eta _2),\ldots ,f_{\mathscr {D}_{\beta }}(\eta _N) \big ]^T$. (To keep the discussion simple, we refer to the harmonic-mean based approximate morphological operators as harmonic followed by the name of the morphological operator that they approximate.) Parameter $\beta$ governs the properties of the filter, which approaches a linear blurring filter in the limit $\beta \rightarrow +\infty$. The non-linearity of the filter increases as $\beta$ decreases. We thus will refer to $\beta$ as the non-linearity parameter. In the limit $\beta \rightarrow 0$, the action of the filters tends to that of the corresponding morphological operators. Using the harmonic dilate and erode operators in a series, we define the harmonic close operator on $N^{\text {d}}\times 1$ vector ${\varvec{d}}$ as

16 $$\begin{aligned} \mathscr {C}_{r,\beta }^{\Omega ^{\text {d}}}({\varvec{d}}) = \big [\varvec{I}_{N^{\text {d}}} \quad \varvec{0}_{N^{\text {d}}\times (N^{\text {a}} + N^\text {s})} \big ] \mathscr {E}_{r,\beta }\Bigg (\mathscr {D}_{r,\beta }\bigg ( \begin{bmatrix} {\varvec{d}} \\ \varvec{0}_{N^\text {s}} \\ \varvec{1}_{N^{\text {a}}} \end{bmatrix} \bigg )\Bigg ), \end{aligned}$$

where $\varvec{I}_{N^{\text {d}}}$ is the identity matrix of size $N^{\text {d}}\times N^{\text {d}}$. More precisely, in expression (16), first we expand the design to the extended domain $\Omega ^{\text {f}}$, then we apply the filter on $\Omega ^{\text {f}}$, and finally we extract the filtered entries of $\Omega ^{\text {d}}$. Finally, we define (cf. Remark 2) the vector $\mathscr {F}({\varvec{d}})$, which holds the element values of $\alpha _h$ in $\Omega ^{\text {d}}$, as

17 $$\begin{aligned} \mathscr {F}({\varvec{d}}) = \varepsilon +(1-\varepsilon )\,\mathscr {C}_{r,\beta }^{\Omega ^{\text {d}}}({\varvec{d}}). \end{aligned}$$

## The optimization problem

The radiated power of the loudspeaker through the output port is the integral of the Poynting vector over the port. In the discretized case, the radiated power becomes

18 $$\begin{aligned} \mathscr {P} = \text {Re}\sum ^{N^{\text {p}}}_{j=1} \overline{u}^{\text {p}}_j\int _{\gamma ^{\text {p}}_j} p = \frac{1}{2}\Re \bigl \{\varvec{x}^*\varvec{B}\varvec{x}\bigr \}, \end{aligned}$$

where the overline denotes complex conjugate, $\varvec{x}$ is the solution to Eq. (10), $\varvec{x}^*$ is the Hermitian transpose of $\varvec{x}$, and, using the same blocking as in Eq. (10),

19 $$\begin{aligned} \varvec{B} = \begin{bmatrix} \varvec{0} &{} \quad ({\varvec{A}}^{\text {pl}})^T &{}\quad \varvec{0} &{}\quad \varvec{0} &{}\quad \varvec{0} \\ {\varvec{A}}^{\text {pl}} &{}\quad \varvec{0} &{}\quad \varvec{0} &{}\quad \varvec{0} &{}\quad \varvec{0} \\ \varvec{0} &{}\quad \varvec{0} &{}\quad \varvec{0} &{} \quad \varvec{0} &{}\quad \varvec{0} \\ \varvec{0} &{} \quad \varvec{0} &{}\quad \varvec{0} &{} \quad {0} &{}\quad {0} \\ \varvec{0} &{} \quad \varvec{0} &{}\quad \varvec{0} &{}\quad {0} &{} \quad {0} \\ \end{bmatrix}. \end{aligned}$$

A suitable way to evaluate the performance of this type of loudspeaker is to assume the loudspeaker to be placed on an infinite floor in an anechoic chamber and measure the *sound pressure level* (SPL) in dB at $1\,\textrm{m}$ in front of the output port, for a given input voltage. Since the SPL is proportional to the logarithm of the radiated power, we base our design objective on this quantity. More precisely, to optimize for a set of frequencies $f_1,\, f_2,\, \ldots ,\, f_m$, we define the objective function

20 $$\begin{aligned} J\left( \mathscr {F}({\varvec{d}})\right) = \sum ^m_{i=1}\ln {\bigg (\mathscr {P}_{f_i}\bigg )}, \end{aligned}$$

where $\mathscr {P}_{f_i}$ is the output power evaluated according expression (18) in the case where $\varvec{x}$ solves governing Eq. (10) for angular frequency $\omega =2\pi f_i$ and physical design $\alpha _h$ with element values $\mathscr {F}({\varvec{d}})$, as defined in expression (17), in $\Omega ^{\text {d}}$. To solve the optimization problem, we use the method of moving asymptotes (MMA) by Svanberg^27^. Since the MMA expects a minimization problem, we write the optimization problem as

21 $$\begin{aligned} \min _{{\varvec{d}}\in \mathscr {A}}\;\; -J\left( \mathscr {F}({\varvec{d}})\right) . \end{aligned}$$

By relaxing the material indicator function optimization, we obtain physical designs with intermediate values. As previously stated, this is undesirable; thus we deal with intermediate values using penalization and filtering. To suppress the intermediate values in ${\varvec{d}}$, we add a quadratic penalty term^28^ to the objective function, which results in the problem

22 $$\begin{aligned} \min _{{\varvec{d}}\in \mathscr {A}}\quad -J\left( \mathscr {F}({\varvec{d}})\right) + \frac{\zeta }{N^{\text {d}}} {\varvec{d}}^T \left( \varvec{1}-{\varvec{d}}\right) , \end{aligned}$$

where $\zeta$ is a positive penalty parameter.

## Sensitivity Analysis

The MMA algorithm requires the gradients of the objective function with respect to design variables. We employ the adjoint variable method because of its efficiency; it computes the full gradients at the cost of (at most) one extra finite element analysis. Here, we present the sensitivity analysis for the radiated power given by expression (18).

Perturbing $\alpha _h$ and using that $\varvec{B}$ is real and symmetric, we obtain a first order perturbation of the radiated power given by

23 $$\begin{aligned} \delta \mathscr {P} = \Re \bigl \{\varvec{x}^*\varvec{B}\, \delta \varvec{x}\bigr \}. \end{aligned}$$

Similarly, the first order perturbation of Eq. (10) is

24 $$\begin{aligned} \delta \!{\varvec{A}}\, \varvec{x} + {\varvec{A}}\, \delta \varvec{x} = \varvec{0}. \end{aligned}$$

Pre-multiplying Eq. (24) with an arbitrary vector ${\varvec{z}}^*$, we obtain

25 $$\begin{aligned} {\varvec{z}}^*\delta \!{\varvec{A}}\, \varvec{x} + {\varvec{z}}^*\!{\varvec{A}}\, \delta \varvec{x} = 0. \end{aligned}$$

Now we select $\varvec{z}$ as the solution to the so-called adjoint equation

26 $$\begin{aligned} {\varvec{A}}^*\! \varvec{z} = \varvec{B} \varvec{x}, \end{aligned}$$

which yields that $\varvec{x}^*\varvec{B}={\varvec{z}}^*\!{\varvec{A}}$, which substituted into expression (23) gives

27 $$\begin{aligned} \delta \mathscr {P} = \Re \bigl \{\varvec{z}^*{\varvec{A}} \delta \varvec{x}\bigr \} = -\Re \bigl \{{\varvec{z}}^*\delta \!{\varvec{A}}\, \varvec{x}\bigr \}, \end{aligned}$$

where last equality follows from expression (25).

Recall that $\alpha _h$ is element-wise constant. Letting $\Omega _n$ be the *n*th element in $\Omega ^{\text {d}}$—so $a_n$ is the element value of $\alpha _h$ in $\Omega _n$—expression (27) implies that

28 $$\begin{aligned} \frac{\partial \mathscr {P}}{\partial a_n} = -\Re \bigl \{{\varvec{z}}^*\varvec{E}^{(n)} \varvec{x}\bigr \}, \end{aligned}$$

where $\varvec{E}^{(n)}$ has entries

29 $$\begin{aligned} e^{(n)}_{ij} = c^2 \int _{\Omega _n} \nabla \varphi _i \cdot \nabla \varphi _j -\omega ^2\int _{\Omega _n} \varphi _i \varphi _j \end{aligned}$$

if $1\le i,j \le N^\Omega$, else $e^{(n)}_{ij} = 0$. Expression (28) provides sensitivities with respect to the physical design $\varvec{a}$, which in turn depends on design variables ${\varvec{d}}$, which are the ones updated by the MMA algorithm, through a compound function involving the non-linear filter, as detailed in expression (17). Using the chain rule, as presented in detail by Hägg and Wadbro^29^ together with expression (28), we obtain the gradient of $\mathscr {P}$ with respect to ${\varvec{d}}$.

## Numerical experiments and results

Consider a loudspeaker box (recall Fig. 3 (left)) with dimensions $w\times h\times d= 80\,\textrm{cm} \times 100\,\textrm{cm} \times 60\,\textrm{cm}$. A baffle of dimensions $w\times d= 80\,\textrm{cm} \times 60\,\textrm{cm}$, containing an 18-inch transducer, separates the upper and the lower box. The lower box has dimensions $w\times h_\text {l}\times d=\, 80\,\textrm{cm} \times 70\,\textrm{cm} \times 60\,\textrm{cm}$. An output port of dimensions $h_{\text {p}}\times d= 30\,\textrm{cm} \times 60\,\textrm{cm}$ is located at the lower left of the loudspeaker. The electromechanical parameters of the transducer, given in Table 1, are typical for a commercial 18-inch driver.

**Table 1:** Electromechanical properties of the 18 inch transducer.

Parameter	Value
Mechanical compliance $C_{\text {ms}}$ (mm/N)	0.22
Moving mass $M_\text {md}$ (g)	150.0
Mechanical resistance $R_{\text {ms}}$ (kg/s)	6.0
*Bl* Factor (N/A)	22.6
Voice coil resistance *R* $(\Omega )$	5.5
Voice coil inductance *L* (mH)	1.5

Two types of numerical experiments are performed. First, the loudspeaker is optimized for single frequencies ranging from 20 to $100\,\textrm{Hz}$. Second, it is optimized for selected frequency bands. The hybrid model is implemented in MATLAB, and all experiments are performed using a finite element discretization with 280 $\times$ 320 square elements, which yields a side length of each element of $2.5\,\textrm{mm}$. With this resolution, we solve for 363,502 unknowns in Eq. (10).

We start the optimization with ${\varvec{d}} =(1,1,\ldots ,1)^T\in \mathbb {R}^{N^{\text {d}}}$ as initial guess, that is, with no solid material inside the design domain $\Omega ^{\text {d}}$. We employ a continuation technique, in which the penalty and non-linearity parameters are gradually increased and decreased, respectively. Initially, the material distribution problem is solved with a very small value of the penalty parameter ($\zeta = 10^{-4}$). The MMA algorithm computes the residual norm of the Karush–Kuhn–Tucker (commonly referred to as the KKT or the first-order optimality) conditions. We increase the penalty parameter and use the previous solution as the initial guess when the KKT residual norm is less than $10^{-2}$ times the initial KKT residual norm. The intermediate values of ${\varvec{d}}$ become expensive as the penalty increases with each step. As a result, when the penalty parameter is sufficiently large, all entries of ${\varvec{d}}$ are essentially 0 or 1 at optimum. For the non-linear filter, we start with a higher value of the non-linearity parameter ($\beta =10$) and decrease the non-linearity parameter whenever we increase the penalty parameter. Throughout the optimization, the relation $\zeta = 10^{-3}\beta ^{-1}$ holds.

To evaluate the performance of the loudspeaker, we use SPL_1 m_. Assuming that the loudspeaker is placed on an infinite sound-hard floor, the quantity SPL_1 m_ is defined as

30 $$\begin{aligned} \text {SPL}_{1\text {m}}= 20\log _{10}\left( \frac{\left| {p}_{\text {1m}}\right| }{p_{o}}\right) , \end{aligned}$$

where $p_{1\text {m}}$ is the pressure at the floor $1\,\textrm{m}$ in front of the output port, and $p_{o}=20\,\mu \text {Pa}$ is the reference pressure amplitude for computing SPL.

Furthermore, we consider the layout with an empty lower box as our reference loudspeaker, which is also the initial guess for the optimization. For all the results, we compare the SPL_1 m_ of all the optimized designs (single as well as multi-frequency optimization) with the SPL_1 m_ of the reference loudspeaker. To compute the SPL_1 m_, we use $V=1\,\textrm{V}$ as the input voltage to the amplifier in all cases.

**Figure 5:**
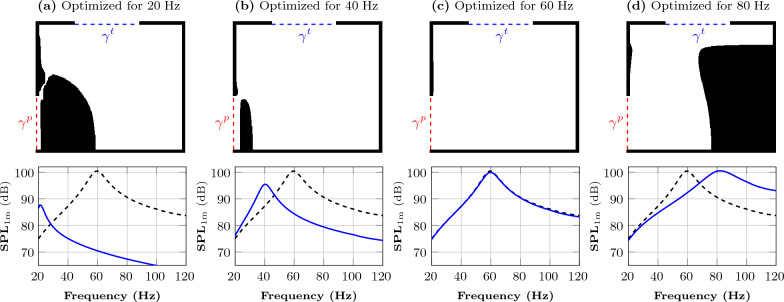
Single-frequency optimization: The figures above the graphs show the material distribution inside the domain $\Omega$ after optimization. The boundary at the top indicates the 18-inch transducer, and the boundary at the lower left is the output port. In the graphs, the dashed black line is the frequency response of the reference loudspeaker (empty lower box), and the solid blue line is the frequency response of the optimized system for the corresponding frequency.

### Single-frequency optimization

The resulting designs and corresponding frequency responses for single-frequency optimizations using target frequencies ranging from 20 to $80\,\textrm{Hz}$ are shown in Fig. 5. The optimized loudspeakers’ frequency response (solid blue line) is compared to the reference loudspeaker’s frequency response (dashed black line). The reference curve has a peak at $60\,\textrm{Hz}$. We note that the frequency response of the optimized design yields a peak at the frequency subject to optimization. Here, the optimization algorithm tunes the resonance frequency of the system to the frequency subject to optimization. Figure 5a,b show that the peak SPL_1 m_ of the designs for the lower target frequencies (20 and 40 $\,\textrm{Hz}$) is lower than the peak SPL_1 m_ of the reference loudspeaker. However, the peak SPL_1 m_ of the designs for the higher target frequency (80 $\,\textrm{Hz}$) is close to that of the reference curve (Fig. 5d). As we already have a peak at $60\,\textrm{Hz}$, the optimization algorithm for this target frequency does not put solid material (Fig. 5c) inside the lower box, and the frequency response overlaps the reference curve. This suggests that the initial design (empty lower box) is a local minimum to the optimization problem for 60 $\textrm{Hz}$. The improvement in the efficiency at the target frequency is between $0\,\textrm{dB}$ (for $60\,\textrm{Hz}$) and $+10\,\textrm{dB}$ (for $80\,\textrm{Hz}$). At the lowest frequency in the range, $20\,\textrm{Hz}$, the peak of frequency response is at $86.5\,\textrm{dB}$. This corresponds to an increase in efficiency of approximately $+11.5\,\textrm{dB}$ at $20\,\textrm{Hz}$ compared to the empty box. Here, the single-frequency optimization is only used for preliminary investigation. The main objective of this study is to perform multi-frequency optimization of the loudspeaker enclosure presented in the following section.

**Figure 6:**
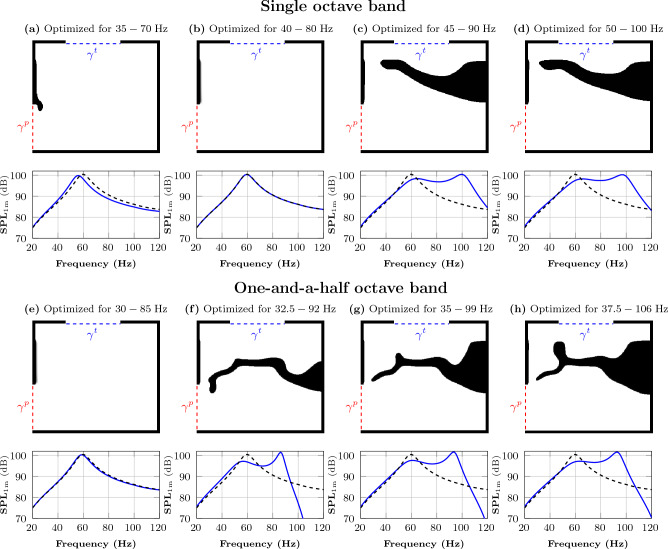
Multi-frequency optimization for single and one-and-a-half octave bands: The figures above the graphs show the material distribution inside the domain $\Omega$ after optimization. The boundary at the top indicates the 18-inch transducer, and the boundary at the lower left is the output port. In the graphs, the dashed black line is the frequency response of the reference loudspeaker (empty lower box), and the solid blue line is the frequency response of the optimized system for the selected frequency range.

### Multi-frequency optimization

Here, we optimize the loudspeaker over single-octave bands, one-and-a-half octave bands, and a double-octave band. The optimization frequencies are logarithmically spaced within the design frequency band. That is, $f_i =F_\text {start}2^{i/12}$, for $i=0, 1, 2, \ldots , n-1$, with $F_\text {start}$ being the lowest frequency in the range, and $n=13$, $n=19$, or $n=26$ if the frequency band is a single octave wide, one-and-a-half octave wide, or a double octave wide, respectively.

For the single octave band optimizations, the frequency bands are 35–70 $\,\textrm{Hz}$, 40–80 $\,\textrm{Hz}$, 45–90 $\,\textrm{Hz}$, and 50–100 $\,\textrm{Hz}$. The optimized loudspeaker designs along with their frequency responses are shown in Fig. 6a–d. The frequency responses in Fig. 6a,b peak at 55 Hz and 60 Hz, respectively for bands 35–70 $\,\textrm{Hz}$ and 40–80 $\,\textrm{Hz}$. There is little improvement in the output power for lower frequencies for frequency band 35–70 $\,\textrm{Hz}$. The frequency response largely overlaps the reference curve for frequency band 40–80 $\,\textrm{Hz}$. Here, the optimization algorithm adds very little solid material inside the lower box; that is, the optimization shows that the empty (initial) design is close to a local minimum for the targeted frequency band. However, for the last two bands, which are, 45–90 $\,\textrm{Hz}$ and 50–100 $\,\textrm{Hz}$, the frequency responses in Fig. 6c,d show that there are improvements in the output power over the full frequency band subject to optimization.

The frequency bands for the one-and-a-half octave band optimization are 30–85 $\,\textrm{Hz}$, 32.5–92 $\,\textrm{Hz}$, 35–99 $\,\textrm{Hz}$, and 37.5–106 $\,\textrm{Hz}$. Fig. 6e–h show the results. The frequency response overlaps the reference curve in Fig. 6e, and there is no improvement in performance. Again, the optimized design is very similar to the initial design, with very little solid material within the design domain. This indicates that the initial design is close to a local minimum. To avoid this minimum, we chose a slightly higher starting frequency and considered the frequency band 32.5–92 $\,\textrm{Hz}$. The frequency response in Fig. 6f demonstrates an improved bandpass design with a larger, more distinct pass band compared to the 30–85 $\,\textrm{Hz}$ band. The results for 35–99 $\,\textrm{Hz}$ and 37.5–106 $\,\textrm{Hz}$, shown in Fig. 6g,h, are qualitatively similar.

**Figure 7:**
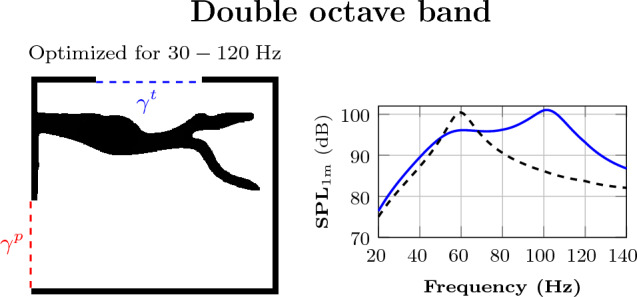
Multi-frequency optimization for double-octave band: Left: Figure shows the material distribution inside the domain $\Omega$ after optimization. The boundary at the top indicates the 18-inch transducer, and the boundary at the lower left is the output port. Right: In the graph, the dashed black line is the frequency response of the reference loudspeaker (empty lower box), and the solid blue line is the frequency response of the optimized system for the selected frequency range.

For the double-octave band optimization, the frequency band is 30–120 $\,\textrm{Hz}$, and the results are shown in Fig. 7. In comparison to the one-and-a-half octave band, the frequency response shows a wider bandpass design and slightly better performance for low frequencies than the reference curve.

Comparing Fig. 6c with 6g and 6d with 6h, the single octave band results look superficially better than the one-and-a-half octave results. This is due to the higher SPL peak achieved inside the target frequency range for the latter, which compensates for the lower levels outside the target frequency band. The one-and-a-half octave band objective function is indeed worse for the single-octave-band design, compared over the corresponding one-and-a-half octave band. Above the design frequency band, the SPL curve can behave unpredictably, which is in analogy with for instance filter design or high order polynomial interpolation.

## Discussion

### Single-frequency optimization

As previously stated, by placing solid material into the design domain, the optimization algorithm tunes the system’s resonance frequency to the frequency under consideration. It should be noted that tuning the resonance frequency to the optimization target frequency is accomplished in two ways, depending on whether the target frequency is below or above the resonance frequency for the reference design. In addition to the results presented in Fig. 5, we performed additional experiments targeting other frequencies. For all our experiments, optimizing for lower frequencies yields a reduction in the port size while optimizing for higher frequencies results in a reduction in the lower box volume.

The lower box acts as a Helmholtz resonator^30^ because the wavelength is large compared to the dimensions of the lower box. The standard lumped model for the resonance frequency of the Helmholtz resonator is $(2\pi )^{-1}c\sqrt{{A_{\text {p}}/(L_{\text {p}}V_\text {l})}}$, where $A_{\text {p}}$ and $L_{\text {p}}$ are the area and effective acoustic length of the output port, respectively, and $V_\text {l}$ is the volume of the lower box. According to Fig. 5, there is more material inside the lower box for 20 Hz as compared to 40 Hz, and reducing the volume of the lower box increases the resonance frequency, and vice versa. It seems that a slightly increased opening could be compensated by a reduction in volume. It should be noted that the single-frequency case tends to invoke extreme designs when the target frequency is pushed toward the physically lower limit. The designs of Fig. 5a,b contain narrow channels which would likely induce nonlinear effects for appreciable sound levels. If, for some reason, a single frequency subwoofer would be of practical interest, a design constraint precluding too narrow channels would be advisable.

### Multi-frequency optimization

To achieve a bandpass design, a dividing wall appears in the lower box for the single octave bands 45–90 $\,\textrm{Hz}$ and 50–100 $\,\textrm{Hz}$, as well as for the one-and-a-half-octave bands 32.5–92 $\,\textrm{Hz}$, 35–99 $\,\textrm{Hz}$, 37.5–106 $\,\textrm{Hz}$, and the double octave-band 30–120 $\,\textrm{Hz}$. The design can be seen as a cascade of two Helmholtz resonators. A lumped model analysis suggests that this loudspeaker box corresponds to a sixth-order acoustic filter with a high-frequency roll-off of 36 $\,\textrm{dB}$ per octave. This is the limit behavior occurring for frequencies well above the design frequency band. The even faster roll-off seen for the presented designs is governed by the quality factor of the Helmholtz resonators. The lumped filter cascade model assumes that the filter elements somehow retain their acoustical identity when connected. This assumption can be far from accurate due to the near field interaction between the resonators in the subwoofer and can be interpreted as one culprit for the failure of the lumped model.

### Validation of the hybrid model

**Figure 8:**
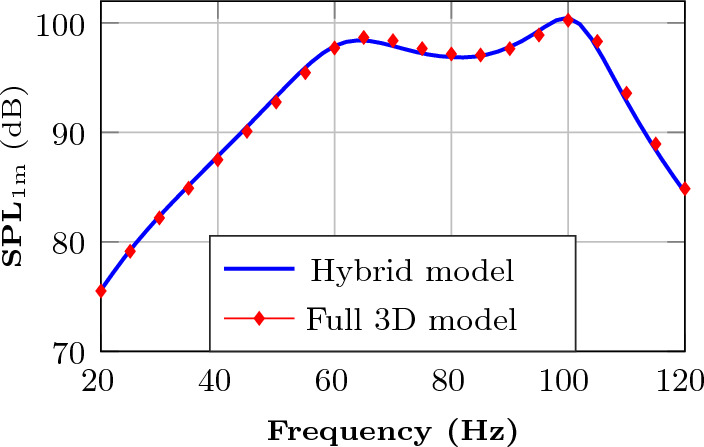
The frequency response for the optimized design in Fig. 6c evaluated using the hybrid model (solid blue line) and the 3D model (red diamonds).

Recall that for computational efficiency, we use a hybrid model, where we assume planar symmetry in the acoustic pressure along the *z*-axis in the lower box. It is reasonable to ask how accurate this assumption is, particularly when solid material is placed inside the lower box. To assess this issue, we consider the design optimized for the one-octave design (45–90 $\,\textrm{Hz}$), depicted in Fig. 6c. For this design, we use COMSOL Multiphysics to implement a full 3D model, as detailed by Bokhari et al.^23^, with second-order tetrahedral elements with a maximum side length of 0.05 m. The 3D model uses a body-conforming mesh and sound-hard boundary conditions at the interfaces between air and solid material in the lower box. Figure 8 shows the loudspeaker’s frequency response computed using the hybrid and the 3D model. This result validates the fidelity of the hybrid model.

## Concluding remarks

In this article, we have introduced a material distribution based topology optimization method for the interior structure of a loudspeaker cabinet. An important development, without which the optimization likely would not be computationally feasible, is the previously reported hybrid 2D–3D model^23^. The presented results are computed for the particular case study of a bandpass subwoofer; note that the final layouts will depend on the parameters of the transducer, the sizes of the front and back chambers, and the targeted frequencies. For the parameters used in this study, the empty box and the optimized designs are topologically equivalent. However, we did not need to impose such a restriction. By using topology optimization, the resulting design may have a different topology than the empty box. That is, the method opens the possibility to find conceptually new designs. Moreover, the methodology is general and can be used to design the interior cabinet also for other use cases.

The obtained layouts are crisp and well-defined; however, to produce a commercially viable device, it would likely be necessary to simplify the design into one containing a few simple flat pieces. By parameterizing a collection of such pieces, additional so-called sizing optimization could be carried out in order to fix these parameters.

For our particular case study, we successfully optimized the interior layout of the cabinet for single as well as multiple frequencies. The following are the key characteristics of the loudspeaker designs presented here:

- In the single-frequency optimization, the optimizer tunes the loudspeaker’s cabinet into a resonator for the corresponding frequency.
- In the multi-frequency optimization, there is very little or no improvement in performance for low frequencies compared to the frequency response of the reference loudspeaker.
- To achieve bandpass designs in the multi-frequency optimization, the optimizer converts the loudspeaker’s cabinet into a cascade of Helmholtz resonators.

## Data Availability

The datasets used and/or analysed during the current study available from the corresponding author on reasonable request.
